# Lipoprotein(a) as an early marker of cardiovascular events in high-risk subjects: insights from the Moli-sani cohort study

**DOI:** 10.3389/fcvm.2025.1571395

**Published:** 2025-07-08

**Authors:** Francesco Gianfagna, Simone Poli, Simona Costanzo, Augusto Di Castelnuovo, Teresa Panzera, Amalia De Curtis, Sara Magnacca, Mariarosaria Persichillo, Lorenzo De Santi, Claudia Cristofani, Daniele Loffredo, Chiara Cerletti, Maria Benedetta Donati, Giovanni de Gaetano, Licia Iacoviello, the Moli-sani Study Investigators, Licia Iacoviello

**Affiliations:** ^1^ Research Center in Epidemiology and Preventive Medicine (EPIMED), Department of Medicine and Surgery, University of Insubria, Varese, Italy; ^2^ Novartis Farma SpA, Milan, Italy; ^3^ Research Unit of Epidemiology and Prevention, IRCCS Neuromed, Pozzilli, Italy; ^4^ Department of Medicine and Surgery, LUM University, Casamassima, Italy

**Keywords:** lipoprotein(a), myocardial infarction, cerebrovascular disorders, cardiovascular risk, MACE, cardiovascular prevention, secondary prevention

## Abstract

**Background and aims:**

Epidemiological studies have revealed the role of lipoprotein(a) [Lp(a)] in the etiopathogenesis of cardiovascular disease (CVD). We analyzed the association between Lp(a) and the risk of a major cardiovascular event in subjects with previous CVD.

**Methods:**

The analysis was conducted on the Moli-sani study population (24,325 individuals aged ≥35 years, recruitment from 2005 to 2010), focusing on subjects with prior CVD. Data from standardized questionnaires and blood pressure, anthropometric, and lab measurements were collected. Lp(a) levels were measured using biobanked samples. The cohort was followed for cardiovascular events. The association between Lp(a) levels and risk of major adverse cardiovascular events was analyzed using Kaplan–Meier and Cox regression models.

**Results:**

In total, 1,284 subjects reported a history of CVD at baseline. The mean ± SD Lp(a) level was 23.3 ± 26.0 mg/dl and 51 subjects (4.0%) had levels ≥90 mg/dl. After a median of 7.3 years, 307 CVD events were recorded and validated. Subjects belonging to the highest Lp(a) level group (≥90 mg/dl) showed a worse trend during early follow-up compared with the lowest level group (<30 mg/dl), with a peak during the first 18 months [hazard ratio (HR) = 3.43, 95% confidence interval (CI): 1.43–8.27]. This increase was higher in subjects with dyslipidemia not treated with statins and those with multiple previous CVD events (HR = 11.0, 95% CI: 1.98–61.1; HR = 25.6, 95% CI: 7.83–83.8).

**Conclusions:**

High Lp(a) levels were associated with an increased risk of early secondary cardiovascular events in individuals with a history of multiple CVDs or non-treated dyslipidemia, suggesting that lipoprotein(a) is a modifiable biomarker that can be measured at different times for CVD risk assessment.

## Highlights

• Lipoprotein(a) [Lp(a)] has potential involvement in cardiovascular disease (CVD).
• We compared Lp(a) levels and the risk of major adverse cardiovascular events in 1,284 subjects with previous CVD.
• Lp(a) is significantly associated with the risk of new events, mainly in the first months.
• Lp(a) is strongly associated with CVD risk in patients without lipid-lowering treatments or multiple events.
• Lp(a) may be a novel biomarker that can be used to monitor cardiovascular risk at different times following a CVD event.

## Introduction

Lipoprotein(a) [Lp(a)] is a low-density lipoprotein (LDL)-like molecule in which apolipoprotein B is covalently linked to apolipoprotein A [apo(a)] by a single disulfide bond (1), and has gained attention in cardiovascular research due to its multifaceted pathogenic roles. Lp(a) exhibits proinflammatory and proatherogenic properties, exerting a significant impact on cardiovascular health (2). Of particular interest is its role as the primary carrier of oxidized phospholipids, which are potent contributors to inflammation and atherogenicity (2, 3).

Genetic and epidemiological studies have found that Lp(a) has a pivotal role in the pathophysiology of cardiovascular diseases (CVDs), suggesting its influence in primary prevention settings and various pathological conditions (4–7). Moreover, high Lp(a) levels are likely to have a primary role in aortic valve disease and are further associated with disease progression (8).

Plasma concentrations of Lp(a) are inherited, and up to 70% of the interindividual variability in Lp(a) levels can be explained by the different number of Kringle IV subtype 2 repeats that are present in the apo(a) gene (9). Variations in plasma Lp(a) levels, spanning an extensive range from 0.1 to over 100 mg/dl, exhibit pronounced disparities across ethic groups. Geographically, median Lp(a) levels vary among European cohorts, and lower levels are generally found in Northern European populations than in Central and Southern populations (10). Notably, populations with Caucasian ancestry manifest skewed frequency distributions, with a prevalence of low Lp(a) levels, a trend that is echoed in both the Northern and Southern areas of Italy (11–14). A recent multicenter epidemiological study in a global population measured Lp(a) levels in a small minority of patients with atherosclerotic cardiovascular disease (ASCVD), with elevated levels found in black, younger, and female patients. Lp(a) levels exceeded the cut-off of 50 mg/dl or 125 nmol/L, which is associated with increased risk, in approximately one-fourth of the patients (15). Several studies have identified Lp(a) as a causal risk factor for CVD, independent of traditional risk factors (8, 16, 17). Despite its established causal role in CVD, there is minimal routine testing of Lp(a), hindering comprehensive cardiovascular risk assessment. In addition, several studies have underscored the association between elevated Lp(a) levels and increased CVD risk in patients who achieve their LDL-C targets or who are taking statin therapy (18).

Joint recommendations by the European Society of Cardiology (ESC) and European Atherosclerosis Society (EAS) advocate for measurement of Lp(a) at least once in a lifetime for the general adult population to enhance cardiovascular risk stratification. Despite these recommendations, Lp(a) testing rates remain low, even among individuals with a family history of CVD or those at elevated risk, likely due to the absence of available targeted treatments. However, Lp(a) testing has been correlated with more intensive preventive treatments and improved clinical outcomes, underlining its value in primary and secondary prevention of CVD (19, 20).

Herein, we evaluated the association between Lp(a) levels and CVD events during follow-up in participants with a previous CVD identified within a general population cohort. The study was conducted using data from the Moli-sani study, a comprehensive cohort evaluation focusing on chronic degenerative diseases and their associated risk factors (21). The results may allow us to advocate for Lp(a) as a potential marker to be used for risk stratification or as a causal risk factor to be targeted with specific interventions in a secondary prevention setting.

## Materials and methods

### Study population

This analysis used data from the Moli-sani study conducted between March 2005 and April 2010. This was a general population cohort involving 24,325 individuals (48% men) aged ≥35 years from the Molise region in Italy. Participants were randomly selected through the city hall registries. The Moli-sani study was approved by the Ethics Committee of the Catholic University in Rome, Italy, and also complied with the Declaration of Helsinki. All participants provided written informed consent for participation. Details of the study have been provided elsewhere (21).

Starting from the initial study sample, a total of 1,320 subjects had a previous CVD at enrollment, including myocardial infarction, peripheral artery disease, angina, revascularization procedures, and cerebrovascular events. A self-reported CVD event had to fulfill one of the following criteria: (i) participant-reported hospital admission date, (ii) reported use of medication for ischemic vascular disease, or (iii) presented medical records indicating a diagnosis of ischemic vascular disease. Among the 1,320 subjects with ASCVD at baseline, 1,284 had data on Lp(a) levels and were included in the analysis (22).

### Data collection

At baseline, trained personnel administered standardized questionnaires and performed instrumental and laboratory tests. The questionnaires gathered data on various aspects, including social status, childhood history, medical and pharmacological history, and lifestyle habits. Trained research personnel performed blood pressure (BP) and anthropometric measurements using standardized methods established during preliminary training sessions (21). Body weight and height were measured with no shoes and only light indoor clothing, and body mass index (BMI) was calculated as kg/m^2^. Blood pressure was measured three times while resting on the non-dominant arm by an automatic device (OMRON-HEM-705CP, Omron, Kyoto, Japan), and the mean of the last two values was considered. Systolic blood pressure ≥140 mmHg, diastolic blood pressure ≥90 mmHg, or the use of antihypertensive agents was considered as hypertension. Participants were classified as non-smokers, former (quit for at least 1 year), or current smokers. Dietary habits were assessed with the Italian EPIC food frequency questionnaire (23), from which total consumed kcal and intake of specific macro- and micronutrients were determined. Adherence to the traditional Mediterranean diet was evaluated using the Mediterranean Diet Score (24). Moderate alcohol intake was defined as a regular consumption of no more than one drink per day for women and no more than two drinks per day for men (24).

Venous blood samples were collected via clean venipuncture between 07:00 and 09:00 a.m. after overnight fasting and refraining from smoking for at least 6 h. Blood glucose, total cholesterol, high-density lipoprotein (HDL) cholesterol, LDL cholesterol, and blood cell count were measured, along with other circulating biomarkers, in fresh samples in the centralized Moli-sani clinical chemistry laboratories. Serum lipids and blood glucose were assayed by enzymatic reaction methods using an automatic analyzer [ILab 350, Instrumentation Laboratory (IL), Milan, Italy]. LDL cholesterol was calculated using Friedewald's formula. Total blood cholesterol ≥240 mg/dl or use of drug treatment was considered a diagnosis of hypercholesterolemia. Lipid-lowering treatment was self-reported (22). Blood glucose ≥126 mg/dl or specific use of antihyperglycemic medication was considered a diagnosis of diabetes. Creatinine levels were measured in serum stored in liquid nitrogen as part of the European BiomarCaRE project. Chronic kidney disease was defined as a creatinine-estimated glomerular filtration rate (eGFR) <60 ml/min/1.73 m^2^. Lp(a) concentrations were assessed in serum samples, stored at the Neuromed Biobanking Center in Pozzilli, using a fully automated, particle-enhanced turbidimetric immunoassay [Biokit Quantia Lp(a)-Test; Abbott Diagnostics, Abbott Park, IL, USA] at the European project BiomarCaRE in Hamburg between 2011 and 2015. This assay is not affected by size heterogeneity (25) and has a detection limit of 0.38 mg/dl over a range of 1.3–90.0 mg/dl. The laboratory maintained high precision in measurements, with an intra-assay coefficient of variation of 2.1% and an inter-assay coefficient of 6.5%. To preserve sample integrity, serum aliquots were stored at −80°C until analysis, ensuring consistency and preventing potential degradation. A single thaw of the aliquots was performed before Lp(a) level determination to minimize the impact of freeze-thaw cycles. Within the BiomarCaRE consortium, no associations were identified between the storage time of single aliquots and Lp(a) levels, further validating the reliability of the measurements (9).

The cohort was followed up for major cardiovascular events (MACE) until 31 December 2015. MACE was defined as the composite endpoint of cardiovascular death, non-fatal stroke, non-fatal acute myocardial infarction, unstable angina, and coronary revascularization. Events that occurred during follow-up were ascertained through hospital discharge files, the regional death Registro Nominativo delle Cause di Morte (ReNCaM) registry, and death certificates [Istituto nazionale di statistica (ISTAT) form], using the International Classification of Diseases, ninth revision (ICD-9).

For coronary heart disease (CHD), ICD-9 codes 410–414 and/or reperfusion procedure (ICD-9 codes 36.0–36.9) were considered. For cerebrovascular disease, ICD-9 codes 430–432, 434, or 436–438, or carotid revascularization procedure codes (ICD-9 code 38.12) were included. Suspected CHD deaths were identified when ICD-9 codes 410–414 or 798 and 799 were listed as the underlying cause of death or when codes 250, 401–405, or 420–429 were reported as the underlying cause with codes 410–414 as a secondary cause. Suspected cerebrovascular deaths were identified when ICD-9 codes 430–438 were recorded as the underlying, antecedent, or direct cause of death. Standardized procedures for epidemiology and clinical research studies were used for validation of all the events (26). Time to event was calculated until the date of the diagnosis of MACE, the date of death, or the date of the last contact prior to December 2015.

### Statistical analysis

Summary statistics for the phenotypes involved reporting observations, missing values, means, and standard deviations for continuous variables. For categorical variables, observations, missing values, and frequency distributions were reported. Statistical tests were used to highlight differences between means and group frequencies, using *t*-tests or Pearson's Chi-square tests based on the type of variable and its distribution and considering an alpha significance level equal to 0.05. The prevalence of subjects with high Lp(a) concentrations, along with 95% confidence intervals (CI), was calculated. As in the HERITAGE study (15), Lp(a) concentration cut-offs were considered categorical variables in the analyses. Incidence of MACE during follow-up was reported using Kaplan–Meier survival curves.

To investigate a possible association between Lp(a) levels and the risk of MACE, a survival analysis and Cox-proportional hazard regression models were used. Adjustment for age as the time scale and sex as the strata was made in a regression model (Model 1). A further statistical model was used considering as covariates the available variables plausibly related to Lp(a) and CVD pathophysiology (see Table 1), which resulted associated with a level of significance *p* < 0.10 with the highest Lp(a) categories (Model 2). Missing variables were analyzed using the complete-case methodology. All the statistical analyses were conducted using SAS 9.4 software (SAS Institute Inc., Cary, NC, USA).

**Table 1:** Characteristics of the population with CVD at baseline.

Variable	All CVD events (*N* = 1,284)			Lp(a) < 30 mg/dl (*n* = 959, 74.7%)		Lp(a) 30–49.9 mg/dl (*n* = 137, 10.7%)		Lp(a) 50–69.9 mg/dl (*n* = 96, 7.5%)		Lp(a) 70–89.9 mg/dl (*n* = 41, 3.2%)		Lp(a) ≥90 mg/dl (*n* = 51, 4.0%)		*p*-value^a^
	NA	Mean	SD	Mean	SD	Mean	SD	Mean	SD	Mean	SD	Mean	SD	
Lp(a) (mg/dl)	0	23.3	26.0	10.5	7.1	39.5	5.7	59.1	6.0	78.7	5.2	>90^b^		—
Age (years)	0	67.3	10.1	67.3	10.1	66.8	10.0	66.8	10.2	66.4	9.4	68.8	10.0	0.92
BMI (kg/m^2^)	6	29.2	4.6	29.1	4.6	29.5	4.4	30.0	4.2	29.5	5.0	29.3	4.5	0.095
Systolic BP (mmHg)	3	149.6	21.2	150.1	21.5	147.6	21.3	147.8	18.2	148.2	16.8	150.2	25	0.36
Diastolic BP (mmHg)	2	80.5	9.9	80.6	10.0	80.6	10.1	81.2	9.0	81.9	9.5	77.2	8.7	0.30
Mediterranean Diet Score	15	4.44	1.66	4.4	1.65	4.39	1.67	4.51	1.8	5.12	1.47	4.6	1.69	0.051
Alcohol intake (g/day)	15	17.3	20.9	17.5	20.8	15.0	19.9	18.1	23.7	19.8	22.0	17.5	19.3	0.90
Food intake (kcal/day)	15	1,870	655	1,874	670	1,855	633	1,891	605	1,928	514	1,743	610	0.39
Total cholesterol (mg/dl)	9	192.1	42.0	190.8	41.8	194.1	43.3	199.6	43.6	192.3	36.7	198.0	42.5	**0.043**
LDL (mg/dl)	29	111.2	34.7	110.1	34.8	112.6	35.2	117.5	35.2	109.4	30.3	116.1	33.3	0.068
HDL (mg/dl)	9	53.0	14.3	52.9	14.2	51.8	13.3	54.4	16	54.6	13.3	54.4	14.5	0.18
Triglycerides (mg/dl)	9	141.7	81.6	141.4	85.4	146.8	72.4	139.9	70.3	141.1	59.0	137.5	67.6	0.80
Glucose (mg/dl)	9	113.7	37.1	114.7	38.3	110.9	30.4	114.8	40.2	104.9	31.4	109.1	25.7	0.092
Glomerular filtration rate (ml/min/1.73 m^2^)	43	80.0	18.4	79.8	18.6	81.6	17.6	83.1	17.2	76.2	20.4	77.0	16.8	0.51
C-reactive protein (mg/L)	0	3.27	3.92	3.19	3.86	3.45	3.66	3.40	4.28	3.00	3.11	4.33	5.38	0.11
Age at first CVD events (years)	152	58.2	11.3	58.5	11.1	57.2	12.2	56.6	11.5	56.3	12.4	60.5	11.2	0.50

		All CVD events (*N* = 1,284)			Lp(a) < 30 mg/dl (*n* = 959, 74.7%)		Lp(a) 30–50 mg/dl (*n* = 137, 10.7%)		Lp(a) 50–70 mg/dl (*n* = 96, 7.5%)		Lp(a) 70–90 mg/dl (*n* = 41, 3.2%)		Lp(a) ≥90 mg/dl (*n* = 51, 4.0%)		*p*-value^a^
		NA	*N*	%	*N*	%	*N*	%	*N*	%	*N*	%	*N*	%	
Age classes (years)	35–59		313	24.4	233	24.3	36	26.3	24	25.0	10	24.4	10	19.6	0.99
	60–69		420	32.7	315	32.8	45	32.8	30	31.2	12	29.3	18	35.3	
	≥70		551	42.9	411	42.9	56	40.9	42	43.8	19	46.3	23	45.1	
Sex	Female		414	32.2	314	32.7	44	32.1	29	30.2	11	26.8	16	31.4	0.93
Smoking status	Never		467	36.4	357	37.3	51	37.2	33	34.4	13	31.7	13	25.5	0.28
	Former		640	49.9	476	49.7	68	49.6	48	50.0	23	56.1	25	49.0	
	Current		176	13.7	125	13.0	18	13.1	15	15.6	5	12.2	13	25.5	
Alcohol in moderation		15	362	28.5	274	28.9	40	29.2	24	25.3	13	31.7	11	22.9	0.82
Chronic kidney disease		43	192	15.5	145	15.7	16	11.9	11	12.1	11	28.8	9	18.8	**0** **.** **15**
Type 2 DM		17	220	17.4	171	18.1	21	15.6	15	15.8	4	9.8	9	17.7	0.67
Hypertension		13	1,103	86.8	823	86.9	117	86.0	84	87.5	37	90.2	42	82.4	0.72
Dyslipidemia		39	720	57.8	498	53.8	82	60.7	69	73.4	30	75.0	41	82.0	**<0** **.** **0001**
Statin treatment		105	517	43.9	357	40.7	54	44.3	48	51.6	28	73.7	30	62.5	**<0** **.** **0001**
Previous CVD	≥2 at baseline	2	371	28.9	260	27.1	47	34.3	30	31.3	21	51.2	13	26.0	**0** **.** **015**

BP, blood pressure; DM, diabetes mellitus; CVD, cardiovascular disease; NA, not available.

^a^ Logistic regression (age and sex as covariates); bold values denote *p* < 0.05.

^b^ Higher than the upper limit of detection (90 mg/dl).

## Results

### Population characteristics

Table 1 reports the characteristics of the 1,284 subjects with a history of CVD at baseline, stratified by Lp(a) categories. The patients had a mean (SD) age of 67.3 (10.1) years, 32.2% were female, 17.4% had diabetes, and 28.9% experienced more than one previous cardiovascular event. The mean (SD) level of Lp(a) was 23.3 (26.0) mg/dl and 51 subjects (4.0%) reported levels ≥90 mg/dl. Total cholesterol levels, dyslipidemia prevalence, and statin treatment were higher in the high Lp(a) level group. The prevalence of subjects with a history of multiple cardiovascular events was higher in the Lp(a) groups with levels between 70 and 90 mg/dl.

### Lp(a) level and risk of MACE in subjects with previous CVD

**Figure 1:**
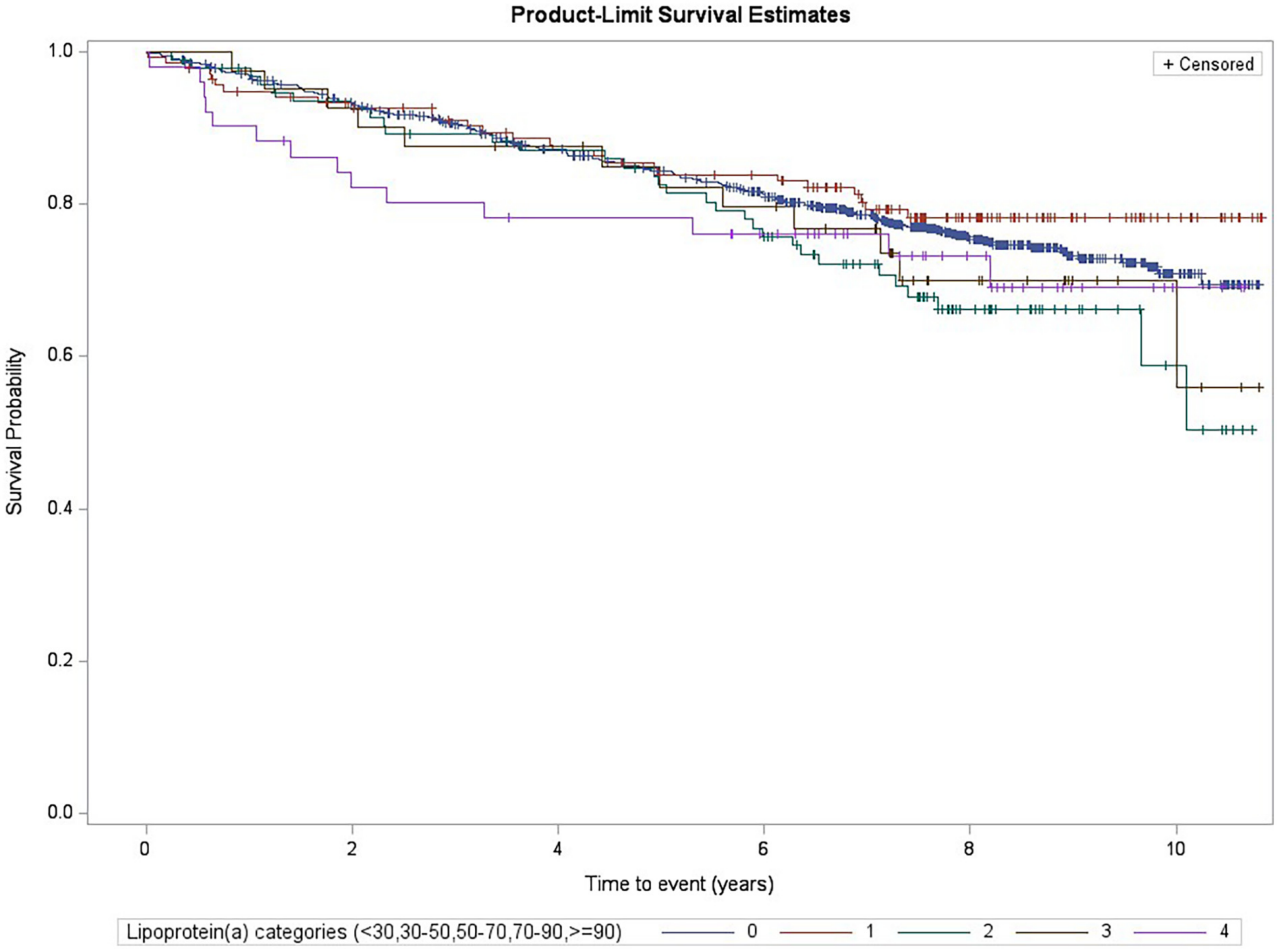
Kaplan–Meier estimates for MACE risk by Lp(a) categories (log-rank test *p* = 0.23, subjects with levels <30 mg/dl were the reference group).

During follow-up (median follow-up of 7.3 years), a total of 307 MACE were recorded and validated. Figure 1 displays the Kaplan–Meier estimates of first-event incidence stratified by Lp(a) category. Although the first four of the five categories showed similar curves, subjects belonging to the highest Lp(a) category (≥90 mg/dl) showed a worse trend during the early follow-up period.

**Figure 2:**
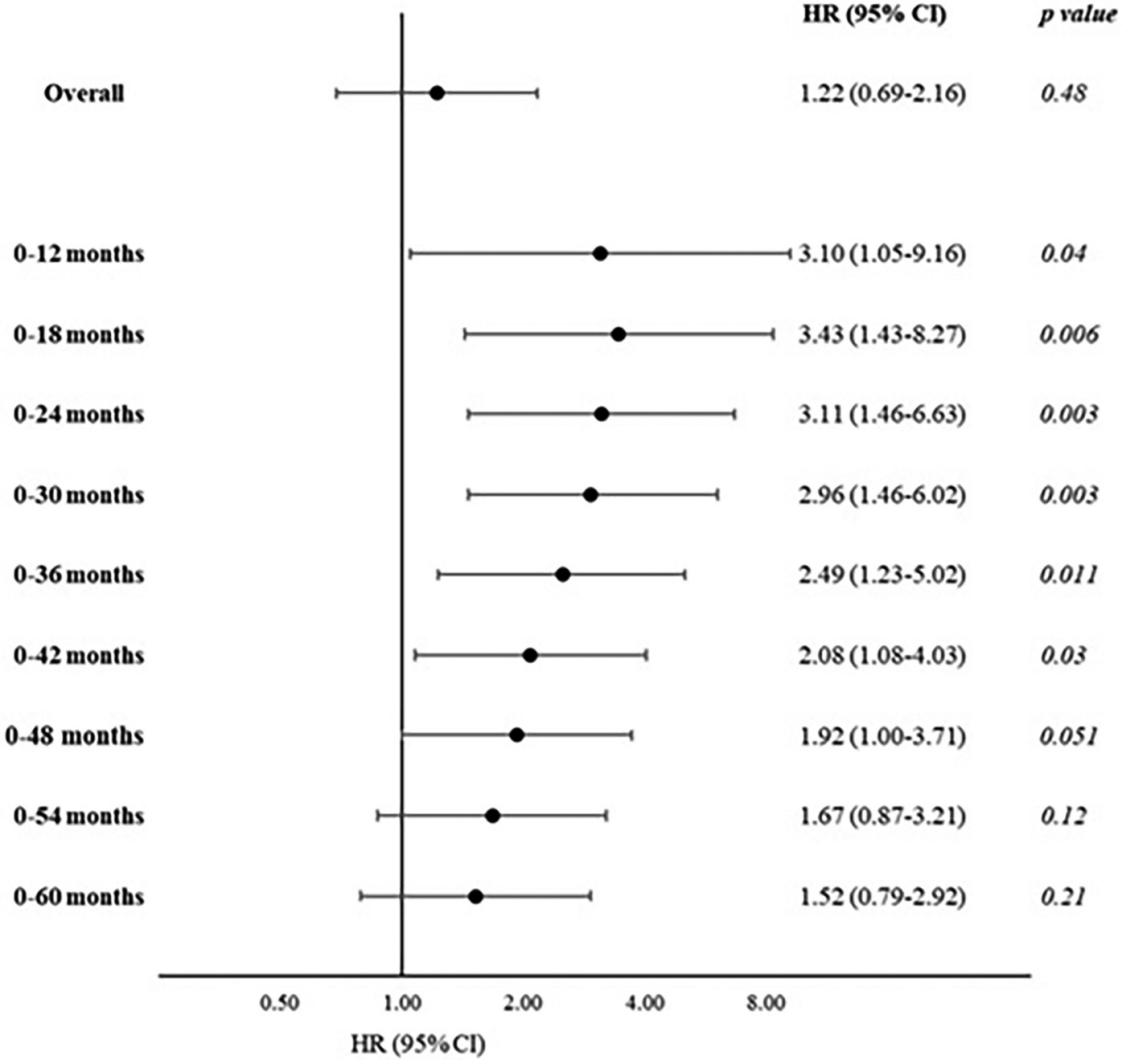
Cox regression model calculating the association between MACE during follow-up and Lp(a) categories: the highest Lp(a) category (≥90 mg/dl) vs. the lowest category (<30 mg/dl), stratified for length of follow-up (increasing from 0 to 12 months, plus 6 months each; age, sex, BMI, Mediterranean Diet Score, food intake, glucose, total cholesterol, and statin treatment).

We performed Cox regression analyses on the association between the Lp(a) categories and MACE. When the MACE incidence among each category was compared with the reference category [subjects with an Lp(a) concentration <30 mg/dl], no statistically significant differences were found. Table 2 shows the results of the Cox regression analysis of the association between the highest Lp(a) category (≥90 mg/dl) and the reference category [overall follow-up period: Model 1, hazard ratio (HR) = 1.21, 95% CI: 0.70–2.08; Model 2, HR = 1.22, 95% CI: 0.69–2.16]. Given the worse trend of the highest Lp(a) category in the early follow-up period observed in the Kaplan–Meier analysis, we performed *a posteriori* analysis, repeating these Cox regression models using different follow-up times and dividing the follow-up at increasing cut-offs (each 6 months). Using Model 1, we observed an increased risk of MACE at early follow-up periods, from 0 to 12 months [HR = 3.02, 95% CI: 1.17–7.81, number of events/subgroup size of 30/223 and 5/14 among the lowest and the highest Lp(a) categories, respectively] up to 0–42 months of follow-up (HR = 1.94, 95% CI: 1.04–3.61, number of events/subgroup size of 110/223 and 11/14, respectively). Similar results were observed using the fully adjusted model (Model 2), with a peak within the first 18 months of follow-up (HR = 3.43, 95% CI: 1.43–8.27) (Table 2 and Figure 2).

**Table 2:** Cox regression model calculating the association between MACE events during follow-up and Lp(a) categories: the highest Lp(a) category (≥90 mg/dl) vs. the lowest category (<30 mg/dl), stratified for length of follow-up (increasing from 0 to 12 months, plus 6 months each).

Follow-up (months)	Model 1				Model 2			
	*n* events; incidence^a^		HR (95% CI)	*p*-value	*n* events; incidence^a^		HR (95% CI)	*p*-value
	<30 mg/dl (*N* = 951)	≥90 mg/dl (*N* = 51)			<30 mg/dl (*N* = 939)	≥90 mg/dl (*N* = 48)		
Overall	223; 34.6	14; 43.6	1.21 (0.70–2.08)	0.49	221; 34.9	13; 42.2	1.22 (0.69–2.16)	0.48
0–12	30; 31.9	5; 103.5	**3.02 (1.17–7.81)**	**0.022**	29; 31.5	4; 87.6	**3.10 (1.05–9.16)**	**0.040**
0–18	45; 32.2	7; 99.1	**2.90 (1.31–6.45)**	**0.009**	44; 32.2	6; 89.6	**3.43 (1.43–8.27)**	**0.006**
0–24	66; 35.9	9; 97.9	**2.57 (1.28–5.16)**	**0.008**	65; 36.1	8; 91.6	**3.11 (1.46–6.63)**	**0.003**
0–30	79; 34.8	10; 89.0	**2.42 (1.25–4.68)**	**0.009**	78; 35.0	9; 84.4	**2.96 (1.46–6.02)**	**0.003**
0–36	90; 33.4	10; 75.6	**2.14 (1.11–4.12)**	**0.022**	89; 33.7	9; 71.6	**2.49 (1.23–5.02)**	**0.011**
0–42	110; 35.5	11; 72.3	**1.94 (1.04–3.61)**	**0.036**	109; 35.9	10; 69.2	**2.08 (1.08–4.03)**	**0.030**
0–48	120; 34.4	11; 64.3	1.77 (0.96–3.29)	0.069	119; 34.8	10; 61.6	1.92 (1.00–3.71)	0.051
0–54	134; 34.6	11; 57.9	1.59 (0.86–2.94)	0.14	133; 35.0	10; 55.4	1.67 (0.87–3.21)	0.12
0–60	145; 34.1	11; 52.6	1.46 (0.79–2.70)	0.22	143; 34.3	10; 50.4	1.52 (0.79–2.92)	0.21

Model 1: age and sex; Model 2: age, sex, body mass index, Mediterranean diet score, food intake, glucose, total cholesterol, statin treatment; in bold *p* < 0.05.

^a^ Per 1,000 person-years.

### Lp(a) level and risk of CVD events in selected subgroups

**Figure 3:**
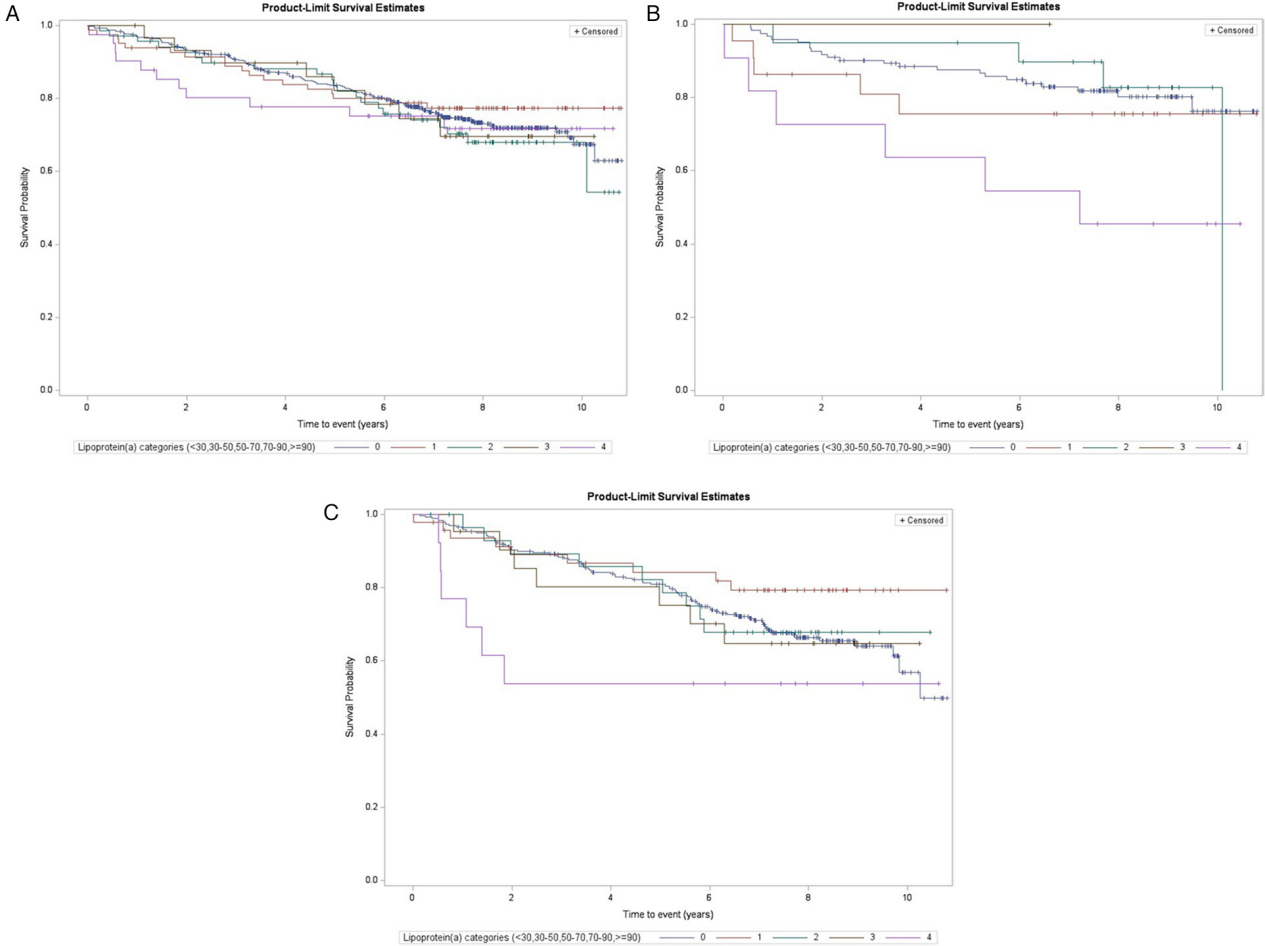
Kaplan–Meier estimates for MACE risk by Lp(a) categories among selected subgroups (log-rank test, subjects with levels <30 mg/dl as reference): **(A)** subjects with dyslipidemia (overall log-rank *p* = 0.86, log-rank *p* for ≥90 mg/dl vs. <30 mg/dl = 0.73), **(B)** subjects with dyslipidemia and without statin treatment (*p* = 0.07 and 0.004), and **(C)** subjects with previous multiple events of CVD (non-fatal stroke, myocardial infarction, or coronary revascularization; *p* = 0.28 and 0.12).

We repeated the analyses in the subgroups of subjects at higher risk, namely those with dyslipidemia without statin treatment and those with multiple previous cardiovascular events. Table 1 shows that these phenotypes were differently distributed among the Lp(a) categories. The Kaplan–Meier curves are reported in Figure 3 and the Cox regression analyses comparing the highest and the lowest (reference) Lp(a) categories (Model 2) are reported in Table 3. In the subgroup of dyslipidemic subjects, the results in the whole follow-up period and at different follow-up times were similar to the whole study population, with a slight increase in risk estimates. In the subgroup of subjects with dyslipidemia and without statin treatment, statistically significant associations were found in the overall follow-up period (Kaplan–Meier log-rank test *p* = 0.004; HR = 4.93, 95% CI: 1.77–13.7) and the increased risk peaked in the first 18–24 months. Finally, in the subgroups of subjects with multiple cardiovascular events at baseline, statistically significant associations were found overall (overall follow-up, HR = 3.95, 95% CI: 1.60–9.73), with very high value in the early follow-up period (at 12 months, HR = 26.3, 95% CI: 5.66–123, number of events/total subjects: 3/11 vs. 9/82) and decreasing risk estimates with increasing follow-up times.

**Table 3:** Cox regression model for the highest Lp(a) category (≥90 mg/dl) vs. the lowest category (<30 mg/dl), stratified for length of follow-up (increasing from 0 to 12 months, plus 6 months each), in selected subgroups.

Follow-up (months)	Dyslipidemia				Dyslipidemia without statin treatment				History of multiple CVD events at baseline			
	Events *N*		HR (95% CI)^a^	*p-*value	Events *N*		HR (95% CI)^a^		Events *N*		HR (95% CI)^a^	*p-*value
	<30 mg/dl, *N* = 491	≥90 mg/dl, *N* = 39			<30 mg/dl, *N* = 122	≥90 mg/dl, *N* = 11			<30 mg/dl, *N* = 253	≥90 mg/dl, *N* = 11		
Overall	127	11	1.34 (0.72–2.50)	0.36	23	6	**4.93 (1.77–13.7)**	**0.002**	82	6	**3.95 (1.60–9.73)**	**0.003**
0–12	15	4	**4.32 (1.37–13.6)**	**0.012**	5	2	**7.41 (1.14–48.1)**	**0.036**	9	3	**26.3 (5.66–123)**	**<0.0001**
0–18	22	6	**4.56 (1.78–11.7)**	**0.002**	5	3	**11.0 (1.98–61.1)**	**0.006**	15	5	**25.6 (7.83–83.8)**	**<0.0001**
0–24	34	8	**4.66 (2.09–10.4)**	**0.0002**	10	3	**7.75 (1.73–34.7)**	**0.007**	25	6	**22.1 (7.89–62.0)**	**<0.0001**
0–30	39	8	**3.86 (1.76–8.50)**	**0.001**	12	3	**5.90 (1.38–25.1)**	**0.016**	27	6	**21.6 (7.79–60.1)**	**<0.0001**
0–36	46	8	**3.08 (1.42–6.67)**	**0.004**	12	3	**5.49 (1.29–23.3)**	**0.021**	30	6	**18.5 (6.78–50.3)**	**<0.0001**
0–42	59	9	**2.54 (1.24–5.21)**	**0.011**	13	4	**6.91 (1.85–25.8)**	**0.004**	37	6	**12.9 (4.92–34.0)**	**<0.0001**
0–48	64	9	**2.31 (1.14–4.71)**	**0.021**	14	4	**5.98 (1.67–21.4)**	**0.006**	41	6	**12.4 (4.74–32.3)**	**<0.0001**
0–54	73	9	1.98 (0.98–4.00)	0.06	15	4	**5.56 (1.58–19.6)**	**0.008**	45	6	**10.1 (3.93–26.1)**	**<0.0001**
0–60	79	9	1.82 (0.90–3.67)	0.09	15	4	**5.56 (1.58–19.6)**	**0.008**	47	6	**8.84 (3.45–22.6)**	**<0.0001**

^a^ Age, sex, body mass index, Mediterranean Diet Score, food intake, glucose, total cholesterol, and (except for subgroup without subjects treated with) statin treatment were covariates; in bold *p* < 0.05.

## Discussion

This study focused on a subgroup within the Moli-sani cohort comprising individuals with documented CVD at baseline. In this population, we found a low prevalence of subjects with high levels of Lp(a) (≥90 mg/dl) as compared with other studies (8, 15). However, these subjects showed an increased risk of MACE during the early follow-up period when compared with subjects with low Lp(a) levels. This outcome was particularly relevant in the dyslipidemic subjects who were not receiving statin treatment, and in those with a history of multiple cardiovascular events. Guided by the approach detailed in the ESC/EAS consensus (8), this research adopted specific cut-offs for Lp(a) concentrations. These cut-offs aimed to delineate values indicating a clinically significant increase in risk, beyond the “rule-out” (<30 mg/dl) and the “rule-in” (>50 mg/dl) thresholds and the intermediary zone (30–50 mg/dl), focusing on those at extreme Lp(a) concentrations, as per the HORIZON clinical trial (NCT04023552).

In our population, up to 75% of the subjects with previous cardiovascular events had an Lp(a) level below 30 mg/dl, while a quarter had levels ≥30 mg/dl and approximately 15% had values ≥50 mg/dl. Lower prevalences were observed for subjects with very high (≥70–89 mg/dl) and extremely high (≥90 mg/dl) Lp(a) levels, with 3.2% and 4.0% of the subjects, respectively. This study population demonstrated demographic and clinical characteristics that slightly diverged from those observed in comparable international studies on Lp(a) in secondary prevention. Specifically, the subjects in this study were younger and included a higher proportion of female participants compared to the HERITAGE study population (15).

Meta-analyses of secondary prevention studies, despite some heterogeneity, demonstrated that elevated Lp(a) levels are an independent predictor of cardiac and cardiovascular events in patients with coronary artery disease (27, 28). These results were further confirmed by recent population studies (29–31) and the AIM-HIGH trial (32). Our result also supports the association between high Lp(a) levels and an increased risk of MACE. This association was stronger during the early follow-up period, within the first 42 months following Lp(a) measurement, and decreased over longer follow-up duration. This result could be due to biomarker levels progressively rising as the event approaches. In fact, the observed values were progressively higher than expected as the measurement time moved closer to the event time. However, a cohort study with repeated measures of Lp(a) is mandatory to confirm this hypothesis. The alternative hypothesis could be that each subject has constant Lp(a) levels, and those with high Lp(a) levels are simply at constant risk of multiple events, which could occur in close succession. The latter could explain the increased probability of early events following measurement of high Lp(a) values. However, in our population, the subjects with the highest Lp(a) levels did not show a high prevalence of previous multiple CVD events. This result did not support this alternative hypothesis. In line with our results, a recent study reported an increased risk of MACE due to high Lp(a) values in individuals with a history of ASCVD during the first year following diagnosis. However, longer follow-up periods were not investigated (30). Assuming that individual Lp(a) levels vary during one’s lifespan, this result suggests that a prolonged wave of elevated Lp(a) levels could mark a period characterized by a high risk of multiple CVD events. Although the majority of the circulating Lp(a) level is genetically determined, concentrations over one’s lifespan could vary due to dietary changes and fluctuations in clinical or subclinical conditions, such as inflammation and the occurrence of cardiovascular events (14, 33–35). Therefore, repeated Lp(a) measurements are suggested for secondary prevention, once the potential clinical utility of Lp(a) as an early marker of an impending MACE is evaluated, as they could potentially indicate a changing health status.

Our subgroup analyses indicated that the prognostic value of Lp(a) is particularly evident in subjects at high risk, such as those with a history of multiple MACE, those with hyperlipidemia, and those with dyslipidemia without treatment with statins. ESC/EAS guidelines for dyslipidemia pay particular attention to patients at a very high risk with recurrent events, recommending a more intensive lipid-lowering treatment approach to control LDL-C with a target of <1.0 mmol/L (<40 mg/dl) (36). Our results highlight the potential clinical application of Lp(a) as a tool for identifying high-risk patients prone to recurrent events and for secondary prevention as an early marker of impending MACE. Lp(a) concentration can be considered a biomarker and a risk factor to identify patients belonging to high-risk categories. Our data also suggest that Lp(a) monitoring plays a significant role in individuals who have not yet initiated statin therapy. Lp(a) level was independent of total cholesterol levels, underscoring the specific role of Lp(a) as a marker for CVD events in these subjects (37). Moreover, the findings from this study support the possibility of better identifying target populations that will benefit the most from Lp(a)-lowering therapies. Further studies in independent cohorts, with repeated measurements of Lp(a) and traditional CVD risk factors, ideally on a yearly basis, will be essential to confirm these findings and assess their predictive value and potential clinical utility.

Some limitations and strengths of the present study warrant consideration. The study population is limited to a specific region in Southern Italy, even if the demographic and clinical characteristics generally align with or only slightly deviate from those observed in other epidemiological studies (15). However, the Moli-sani population is similar to other population study cohorts studied in Italy. The final, main limitation of this study is the limited number of subjects in the specific subgroup analyses, leading to uncertainty in the generalizability of the results. Given the internal concordance of the results and their biological plausibility, we suggest replicating these analyses in cohort studies with large sample sizes.

The relevance of the potential clinical impact of the findings is a strength of this study. Beyond the association between Lp(a) level and increased risk in subjects with a previous CVD event, the decreasing risk over time since the initial measurement suggests the need for repeated Lp(a) assessments at different intervals to monitor the risk more effectively. Currently, guidelines recommend measuring Lp(a) only once in a patient’s lifetime (36).

Lp(a) is a promising marker for CVD risk assessment and, at the same time, serves as a target for risk-lowering strategies, particularly in high-risk subgroups, such as individuals with a history of CVD events. Its causal role in cardiovascular pathogenesis is supported by recent studies on its association with the progression of coronary atherosclerotic plaque and calcific aortic valve stenosis (38, 39). Given the relatively low cost of the test and the strength of the observed association with early CVD events following its measurement, Lp(a) is emerging as a modifiable, time-dependent biomarker that could soon be included in CVD risk algorithms to monitor the risk of early CVD events in high-risk subjects during their follow-up.

## Data Availability

The data underlying this article will be shared on reasonable request to the corresponding author. The data are stored in an institutional repository (https://repository.neuromed.it) and access is restricted by the ethical approvals and the legislation of the European Union.

## Moli-sani study investigators

The enrollment phase of the Moli-sani Study was conducted at the Research Laboratories of the Catholic University in Campobasso (Italy). The follow-up of the Moli-sani cohort is being conducted at the Research Unit of Epidemiology and Prevention of the IRCCS Neuromed, Pozzilli, Italy.

Steering Committee: Licia Iacoviello (Chairperson), Research Unit of Epidemiology and Prevention, IRCCS Neuromed, Pozzilli, Italy, and Department of Medicine and Surgery, LUM University “Giuseppe Degennaro”, Casamassima, Italy; Giovanni de Gaetano, Research Unit of Epidemiology and Prevention, IRCCS Neuromed, Pozzilli, Italy; Maria Benedetta Donati, Research Unit of Epidemiology and Prevention, IRCCS Neuromed, Pozzilli, Italy.

Scientific Secretariat: Chiara Cerletti (Coordinator), Research Unit of Epidemiology and Prevention, IRCCS Neuromed, Pozzilli, Italy; Marialaura Bonaccio, Research Unit of Epidemiology and Prevention, IRCCS Neuromed, Pozzilli, Italy; Americo Bonanni, Research Unit of Epidemiology and Prevention, IRCCS Neuromed, Pozzilli, Italy; Simona Costanzo, Research Unit of Epidemiology and Prevention, IRCCS Neuromed, Pozzilli, Italy, and Department of Medicine and Surgery, University of Insubria, Varese, Italy; Amalia De Curtis, Research Unit of Epidemiology and Prevention, IRCCS Neuromed, Pozzilli, Italy; Augusto Di Castelnuovo, Research Unit of Epidemiology and Prevention, IRCCS Neuromed, Pozzilli, Italy; Alessandro Gialluisi, Research Unit of Epidemiology and Prevention, IRCCS Neuromed, Pozzilli, Italy, and Department of Medicine and Surgery, LUM University “Giuseppe Degennaro,” Casamassima, Italy; Francesco Gianfagna, Department of Medicine and Surgery, University of Insubria, Varese, Italy; Mariarosaria Persichillo, Research Unit of Epidemiology and Prevention, IRCCS Neuromed, Pozzilli, Italy.

Safety and Ethics Committee: Jos Vermylen, Catholic University, Leuven, Belgio (Chairperson); Renzo Pegoraro, Pontificia Accademia per la Vita, Roma, Italy; Antonio G. Spagnolo, Catholic University, Roma, Italy.

External Event Adjudicating Committee: Deodato Assanelli, Brescia, Italy; Livia Rago, Campobasso, Italy.

Baseline and Follow-up Data Management: Simona Costanzo (Coordinator), Research Unit of Epidemiology and Prevention, IRCCS Neuromed, Pozzilli, Italy, and Department of Medicine and Surgery, University of Insubria, Varese, Italy; Sabatino Orlandi, Research Unit of Epidemiology and Prevention, IRCCS Neuromed, Pozzilli, Italy; Teresa Panzera, Research Unit of Epidemiology and Prevention, IRCCS Neuromed, Pozzilli, Italy.

Data Analysis: Augusto Di Castelnuovo (Coordinator), Research Unit of Epidemiology and Prevention, IRCCS Neuromed, Pozzilli, Italy; Marialaura Bonaccio, Research Unit of Epidemiology and Prevention, IRCCS Neuromed, Pozzilli, Italy; Francesca Bracone, Research Unit of Epidemiology and Prevention, IRCCS Neuromed, Pozzilli, Italy; Simona Costanzo, Research Unit of Epidemiology and Prevention, IRCCS Neuromed, Pozzilli, Italy, and Department of Medicine and Surgery, University of Insubria, Varese, Italy; Giuseppe Di Costanzo, Research Unit of Epidemiology and Prevention, IRCCS Neuromed, Pozzilli, Italy; Simona Esposito, Research Unit of Epidemiology and Prevention, IRCCS Neuromed, Pozzilli, Italy; Alessandro Gialluisi, Research Unit of Epidemiology and Prevention, IRCCS Neuromed, Pozzilli, Italy, and Department of Medicine and Surgery, LUM University “Giuseppe Degennaro,” Casamassima, Italy; Anwal Ghulam, Department of Medicine and Surgery, University of Insubria, Varese, Italy; Francesco Gianfagna, Department of Medicine and Surgery, University of Insubria, Varese, Italy; Martina Morelli, Research Unit of Epidemiology and Prevention, IRCCS Neuromed, Pozzilli, Italy, and Fondazione Veronesi—Piattaforma UMBERTO; Maria Loreto Muñoz Venegas, Research Unit of Epidemiology and Prevention, IRCCS Neuromed, Pozzilli, Italy, and Fondazione Veronesi—Piattaforma UMBERTO; Antonietta Pepe, Research Unit of Epidemiology and Prevention, IRCCS Neuromed, Pozzilli, Italy; Emilia Ruggiero, Research Unit of Epidemiology and Prevention, IRCCS Neuromed, Pozzilli, Italy, and Fellow of the Fondazione Umberto Veronesi, Italy.

Biobank, Molecular and Genetic Laboratory: Amalia De Curtis (Coordinator), Research Unit of Epidemiology and Prevention, IRCCS Neuromed, Pozzilli, Italy; Concetta Civitillo, Research Unit of Epidemiology and Prevention, IRCCS Neuromed, Pozzilli, Italy, and Fondazione Veronesi—Piattaforma UMBERTO; Alisia Cretella, Research Unit of Epidemiology and Prevention, IRCCS Neuromed, Pozzilli, Italy, and Fondazione Veronesi—Piattaforma UMBERTO; Sara Magnacca, Research Unit of Epidemiology and Prevention, IRCCS Neuromed, Pozzilli, Italy.

Recruitment Staff: Mariarosaria Persichillo (Coordinator), Research Unit of Epidemiology and Prevention, IRCCS Neuromed, Pozzilli, Italy; Francesca Bracone, Research Unit of Epidemiology and Prevention, IRCCS Neuromed, Pozzilli, Italy; Giuseppe Di Costanzo, Research Unit of Epidemiology and Prevention, IRCCS Neuromed, Pozzilli, Italy; Martina Morelli, Research Unit of Epidemiology and Prevention, IRCCS Neuromed, Pozzilli, Italy, and Fondazione Veronesi—Piattaforma UMBERTO.

Communication and Press Office: Americo Bonanni, Research Unit of Epidemiology and Prevention, IRCCS Neuromed, Pozzilli, Italy.

Regional Institutions: Direzione Generale per la Salute—Regione Molise; Azienda Sanitaria Regionale del Molise (ASReM, Italy); Agenzia Regionale per la Protezione Ambientale del Molise (ARPA Molise, Italy); Molise Dati Spa (Campobasso, Italy); Offices of Vital Statistics of the Molise region.

Hospitals: Presidi Ospedalieri ASReM: Ospedale A Cardarelli, Campobasso; Ospedale F Veneziale, Isernia; Ospedale San Timoteo,Termoli (CB); Ospedale Ss. Rosario, Venafro (IS); Ospedale Vietri, Larino (CB); Ospedale San Francesco Caracciolo, Agnone (IS); Casa di Cura Villa Maria, Campobasso; Responsible Research Hospital, Campobasso; IRCCS Neuromed, Pozzilli (IS).

A list of the Moli-sani study’s past investigators is available at https://www.moli-sani.org/?page_id=173.

## References

[1] Tsimikas S. A test in context: lipoprotein(a): diagnosis, prognosis, controversies, and emerging therapies. J Am Coll Cardiol. (2017) 69(6):692–711. 10.1016/j.jacc.2016.11.042 28183512

[2] Tsimikas S Brilakis ES Miller ER McConnell JP Lennon RJ Kornman KS Oxidized phospholipids, Lp(a) lipoprotein, and coronary artery disease. N Engl J Med. (2005) 353(1):46–57. 10.1056/NEJMoa043175 16000355

[3] Deb A Caplice NM. Lipoprotein(a): new insights into mechanisms of atherogenesis and thrombosis. Clin Cardiol. (2004) 27(5):258–64. 10.1002/clc.4960270503 15188938 PMC6654090

[4] Clarke R Peden JF Hopewell JC Kyriakou T Goel A Heath SC Genetic variants associated with Lp(a) lipoprotein level and coronary disease. N Engl J Med. (2009) 361(26):2518–28. 10.1056/NEJMoa0902604 20032323

[5] Craig WY Neveux LM Palomaki GE Cleveland MM Haddow JE. Lipoprotein(a) as a risk factor for ischemic heart disease: metaanalysis of prospective studies. Clin Chem. (1998) 44(11):2301–6. 9799757

[6] Patel AP Wang M Pirruccello JP Ellinor PT Ng K Kathiresan S Lp(a) (lipoprotein[a]) concentrations and incident atherosclerotic cardiovascular disease: new insights from a large national biobank. Arterioscler Thromb Vasc Biol. (2021) 41(1):465–74. 10.1161/ATVBAHA.120.315291 33115266 PMC7769893

[7] Langsted A Nordestgaard BG Kamstrup PR. Elevated lipoprotein(a) and risk of ischemic stroke. J Am Coll Cardiol. (2019) 74(1):54–66. 10.1016/j.jacc.2019.03.524 31272552

[8] Kronenberg F Mora S Stroes ESG Ference BA Arsenault BJ Berglund L Lipoprotein(a) in atherosclerotic cardiovascular disease and aortic stenosis: a European atherosclerosis society consensus statement. Eur Heart J. (2022) 43(39):3925–46. 10.1093/eurheartj/ehac361 36036785 PMC9639807

[9] Mukamel RE Handsaker RE Sherman MA Barton AR Zheng Y McCarroll SA Protein-coding repeat polymorphisms strongly shape diverse human phenotypes. Science. (2021) 373(6562):1499–505. 10.1126/science.abg8289 34554798 PMC8549062

[10] Waldeyer C Makarova N Zeller T Schnabel RB Brunner FJ Jorgensen T Lipoprotein(a) and the risk of cardiovascular disease in the European population: results from the BiomarCaRE consortium. Eur Heart J. (2017) 38(32):2490–8. 10.1093/eurheartj/ehx166 28449027 PMC5837491

[11] Fogacci F Cicero AF D'Addato S D'Agostini L Rosticci M Giovannini M Serum lipoprotein(a) level as long-term predictor of cardiovascular mortality in a large sample of subjects in primary cardiovascular prevention: data from the Brisighella Heart Study. Eur J Intern Med. (2017) 37:49–55. 10.1016/j.ejim.2016.08.018 27553697

[12] Noto D Barbagallo CM Cavera G Caldarella R Marino G Pace A Lipoprotein(A) levels and apoprotein(a) phenotypes in a Sicilian population. Ann Ital Med Int. (1998) 13(4):205–8. 10400464

[13] Willeit J Kiechl S Santer P Oberhollenzer F Egger G Jarosch E Lipoprotein(a) and asymptomatic carotid artery disease. Evidence of a prominent role in the evolution of advanced carotid plaques: the Bruneck Study. Stroke. (1995) 26(9):1582–7. 10.1161/01.str.26.9.1582 7660402

[14] Arnold N Blaum C Gossling A Brunner FJ Bay B Ferrario MM C-reactive protein modifies lipoprotein(a)-related risk for coronary heart disease: the BiomarCaRE project. Eur Heart J. (2024) 45(12):1043–54. 10.1093/eurheartj/ehad867 38240386

[15] Nissen SE Wolski K Cho L Nicholls SJ Kastelein J Leitersdorf E Lipoprotein(a) levels in a global population with established atherosclerotic cardiovascular disease. Open Heart. (2022) 9(2):e002060. 10.1136/openhrt-2022-002060 36252994 PMC9577925

[16] Tsimikas S Hall JL. Lipoprotein(a) as a potential causal genetic risk factor of cardiovascular disease: a rationale for increased efforts to understand its pathophysiology and develop targeted therapies. J Am Coll Cardiol. (2012) 60(8):716–21. 10.1016/j.jacc.2012.04.038 22898069

[17] Nordestgaard BG Langsted A. Lipoprotein (a) as a cause of cardiovascular disease: insights from epidemiology, genetics, and biology. J Lipid Res. (2016) 57(11):1953–75. 10.1194/jlr.R071233 27677946 PMC5087876

[18] Bhatia HS Hurst S Desai P Zhu W Yeang C. Lipoprotein(a) testing trends in a large academic health system in the United States. J Am Heart Assoc. (2023) 12(18):e031255. 10.1161/JAHA.123.031255 37702041 PMC10547299

[19] Kronenberg F. Lipoprotein(a) measurement issues: are we making a mountain out of a molehill? Atherosclerosis. (2022) 349:123–35. 10.1016/j.atherosclerosis.2022.04.008 35606072

[20] Sturzebecher PE Schorr JJ Klebs SHG Laufs U. Trends and consequences of lipoprotein(a) testing: cross-sectional and longitudinal health insurance claims database analyses. Atherosclerosis. (2023) 367:24–33. 10.1016/j.atherosclerosis.2023.01.014 36764050

[21] Di Castelnuovo A Costanzo S Persichillo M Olivieri M de Curtis A Zito F Distribution of short and lifetime risks for cardiovascular disease in Italians. Eur J Prev Cardiol. (2012) 19(4):723–30. 10.1177/1741826711410820 21571772

[22] Bonaccio M Di Castelnuovo A Costanzo S Persichillo M De Curtis A Cerletti C Interaction between Mediterranean diet and statins on mortality risk in patients with cardiovascular disease: findings from the Moli-sani study. Int J Cardiol. (2019) 276:248–54. 10.1016/j.ijcard.2018.11.117 30527993

[23] Pala V Sieri S Palli D Salvini S Berrino F Bellegotti M Diet in the Italian EPIC cohorts: presentation of data and methodological issues. Tumori J. (2003) 89(6):594–607. 10.1177/030089160308900603 14870824

[24] Trichopoulou A Costacou T Bamia C Trichopoulos D. Adherence to a Mediterranean diet and survival in a Greek population. N Engl J Med. (2003) 348(26):2599–608. 10.1056/NEJMoa025039 12826634

[25] Simo JM Camps J Gomez F Ferre N Joven J. Evaluation of a fully-automated particle-enhanced turbidimetric immunoassay for the measurement of plasma lipoprotein(a). Population-based reference values in an area with low incidence of cardiovascular disease. Clin Biochem. (2003) 36(2):129–34. 10.1016/s0009-9120(02)00416-2 12633762

[26] Di Castelnuovo A Bonaccio M Costanzo S De Curtis A Persichillo M Panzera T The Moli-sani risk score, a new algorithm for measuring the global impact of modifiable cardiovascular risk factors. Int J Cardiol. (2023) 389:131228. 10.1016/j.ijcard.2023.131228 37527754

[27] O'Donoghue ML Morrow DA Tsimikas S Sloan S Ren AF Hoffman EB Lipoprotein(a) for risk assessment in patients with established coronary artery disease. J Am Coll Cardiol. (2014) 63(6):520–7. 10.1016/j.jacc.2013.09.042 24161323 PMC3945105

[28] Wang Z Zhai X Xue M Cheng W Hu H. Prognostic value of lipoprotein (a) level in patients with coronary artery disease: a meta-analysis. Lipids Health Dis. (2019) 18(1):150. 10.1186/s12944-019-1092-6 31286992 PMC6615167

[29] Madsen CM Kamstrup PR Langsted A Varbo A Nordestgaard BG. Lipoprotein(a)-lowering by 50 mg/dl (105 nmol/L) may be needed to reduce cardiovascular disease 20% in secondary prevention: a population-based study. Arterioscler Thromb Vasc Biol. (2020) 40(1):255–66. 10.1161/ATVBAHA.119.312951 31578080

[30] Welsh P Al Zabiby A Byrne H Benbow HR Itani T Farries G Elevated lipoprotein(a) increases risk of subsequent major adverse cardiovascular events (MACE) and coronary revascularisation in incident ASCVD patients: a cohort study from the UK biobank. Atherosclerosis. (2024) 389:117437. 10.1016/j.atherosclerosis.2023.117437 38219651

[31] Boffa MB Stranges S Klar N Moriarty PM Watts GF Koschinsky ML. Lipoprotein(a) and secondary prevention of atherothrombotic events: a critical appraisal. J Clin Lipidol. (2018) 12(6):1358–66. 10.1016/j.jacl.2018.08.012 30316749

[32] Albers JJ Slee A O'Brien KD Robinson JG Kashyap ML Kwiterovich PO Jr Relationship of apolipoproteins A-1 and B, and lipoprotein(a) to cardiovascular outcomes: the AIM-HIGH trial (atherothrombosis intervention in metabolic syndrome with low HDL/high triglyceride and impact on global health outcomes). J Am Coll Cardiol. (2013) 62(17):1575–9. 10.1016/j.jacc.2013.06.051 23973688 PMC3800510

[33] Maeda S Abe A Seishima M Makino K Noma A Kawade M. Transient changes of serum lipoprotein(a) as an acute phase protein. Atherosclerosis. (1989) 78(2–3):145–50. 10.1016/0021-9150(89)90218-9 2476992

[34] Lampsas S Xenou M Oikonomou E Pantelidis P Lysandrou A Sarantos S Lipoprotein(a) in atherosclerotic diseases: from pathophysiology to diagnosis and treatment. Molecules. (2023) 28(3):969. 10.3390/molecules28030969 36770634 PMC9918959

[35] Harb T Ziogos E Blumenthal RS Gerstenblith G Leucker TM. Intra-individual variability in lipoprotein(a): the value of a repeat measure for reclassifying individuals at intermediate risk. Eur Heart J Open. (2024) 4(5):oeae064. 10.1093/ehjopen/oeae064 39219855 PMC11365507

[36] Mach F Baigent C Catapano AL Koskinas KC Casula M Badimon L 2019 ESC/EAS guidelines for the management of dyslipidaemias: lipid modification to reduce cardiovascular risk. Eur Heart J. (2020) 41(1):111–88. 10.1093/eurheartj/ehz455 31504418

[37] Bjornson E Adiels M Taskinen MR Burgess S Chapman MJ Packard CJ Lipoprotein(a) is markedly more atherogenic than LDL: an apolipoprotein B-based genetic analysis. J Am Coll Cardiol. (2024) 83(3):385–95. 10.1016/j.jacc.2023.10.039 38233012 PMC7616706

[38] Nurmohamed NS Gaillard EL Malkasian S de Groot RJ Ibrahim S Bom MJ Lipoprotein(a) and long-term plaque progression, low-density plaque, and pericoronary inflammation. JAMA Cardiol. (2024) 9(9):826–34. 10.1001/jamacardio.2024.1874 39018040 PMC11255968

[39] Arsenault BJ Loganath K Girard A Botezatu S Zheng KH Tzolos E Lipoprotein(a) and calcific aortic valve stenosis progression: a systematic review and meta-analysis. JAMA Cardiol. (2024) 9(9):835–42. 10.1001/jamacardio.2024.1882 39018080 PMC11255972

